# Functional neutralization of anti-IFN-γ autoantibody in patients with nontuberculous mycobacteria infection

**DOI:** 10.1038/s41598-019-41952-1

**Published:** 2019-04-05

**Authors:** Dyah Ika Krisnawati, Yung-Ching Liu, Yuarn-Jang Lee, Yun-Ting Wang, Chia-Ling Chen, Po-Chun Tseng, Chiou-Feng Lin

**Affiliations:** 1 Graduate Institute of Medical Sciences, College of Medicine, Taipei Medical University, Taipei, 110 Taiwan; 2 Department of Microbiology and Immunology, School of Medicine, College of Medicine, Taipei Medical University, Taipei, 110 Taiwan; 3 Dharma Husada Nursing Academy, Kediri, East Java Indonesia; 4 Department of Internal Medicine, School of Medicine, College of Medicine, Taipei Medical University, Taipei, 110 Taiwan; 5 Division of Infectious Diseases, Department of Internal Medicine, Shuang Ho Hospital, Taipei Medical University, Taipei, 110 Taiwan; 6 Division of Infectious Diseases, Department of Internal Medicine, Taipei Medical University Hospital, Taipei, 110 Taiwan; 7 School of Respiratory Therapy, College of Medicine, Taipei Medical University, Taipei, 110 Taiwan

## Abstract

Interferon (IFN)-γ is crucial for normal immune surveillance and exhibits immunomodulatory, antimicrobial, and anticancer activity. Patients with nontuberculous mycobacteria (NTM) infection commonly express high levels of anti-IFN-γ autoantibodies (autoAbs) and suffer from recurrent infections due to adult-onset immunodeficiency with defects in IFN-γ immune surveillance. In this study, we developed the methods for determination of anti-IFN-γ autoAbs and then characterized their neutralizing activity in patients with NTM infection. A modified sandwich ELISA-based colorimetric assay followed by immunoblot analysis detected the presence of autoAbs in three out of five serum samples. Serum levels of IFN-γ were decreased. Synthetic peptide binding assay showed variable patterns of epitope recognition in patients positive for anti-IFN-γ autoAbs. Functional tests confirmed that patient serum blocked IFN-γ-activated STAT1 activation and IRF1 transactivation. Furthermore, IFN-γ-regulated inflammation, chemokine production and cytokine production were also blocked. These results provide potentially useful methods to assay anti-IFN-γ autoAbs and to characterize the effects of neutralizing autoAbs on IFN-γ signaling and bioactivity.

## Introduction

Type II interferon (IFN)-γ is produced by T cells or natural killer cells in response to stimulation with antigens and/or cytokines. IFN-γ facilitates immune surveillance and has immunomodulatory effects: (1) induction of MHC-associated antigen presentation pathways; (2) development of Th1 responses; (3) anti-microbial activities; (4) stimulation anticancer activities; (5) regulation of leukocyte trafficking; and (6) facilitation of inflammation^1–3^. In the case of anti-bacterial effects, IFN-γ acts as a macrophage activator and promotes innate defense via receptor-mediated phagocytosis enhancing microbicidal effects^1,4^. In general, IFN-γ activates NADPH-dependent phagocyte oxidase system (respiratory burst) through transcriptional induction of gp91^phox^ and p67^phox^ priming for nitric oxide production, tryptophan depletion, and up-regulation of lysosomal enzymes promoting microbe destruction^1^. Immunity-regulated GTPase (IRG) proteins, known as p47 GTPases, are the key mediators of immune surveillance control of IFN-γ against intracellular infection of pathogens and mycobacteria in particular^5,6^. IFN-γ increases expression of IRG proteins to regulate the processing of pathogen-containing vacuoles, particularly in autophagy, stimulating immune defenses of the host cells. Without IFN-γ and/or IRG proteins, host cells are susceptible to mycobacterial infection.

Adult-onset immunodeficiency is currently reported in patients who show defects in IFN-γ signaling commonly resulting from the generation of anti-IFN-γ autoantibodies (autoAbs) and partly due to inherited mutations in the IFN-γ signaling-associated factors; the syndrome appeared for the first time in 2004 in Thailand and Taiwan^7^. In the following studies^7–10^, the generation of anti-IFN-γ autoAbs in infections with tuberculosis, non-tuberculous mycobacteria (NTM), *Cryptococcus neoformans*, *Penicillium marneffei*, and non-typhoidal *Salmonella spp*. are frequently identified, confirming that IFN-γ is a potent anti-tuberculosis, anti-bacterial and immunomodulatory cytokine^11^. This immunosuppressive disorder appear to be chronic and non-contagious affecting mainly people aged around 50 years^10^. Limited publications explored the main pathogenic processes causing the disease. Genetic factors are partly suspected, but the disease does not appear to be inheritable. Additionally, environmental stimuli may trigger the disease including infections and toxins^7^. Major epitope region (amino acid residues 121–131 of human IFN-γ) is crucial for IFN-γ receptor (IFN-γR) activation and the epitope is highly homologous to a stretch of the Noc2 protein of *Aspergillus spp*. which can cross-react with anti-IFN-γ autoAbs from the patients^12^. A molecular mimicry autoimmunity model explaining generation of anti-IFN-γ autoAbs was suggested.

IFN-γ binds to a distinct IFN-γR composed of two subunits, IFNGR1 and IFNGR2, which are associated with Janus kinases (JAK) 1 and JAK2, respectively. Activation of JAKs results in tyrosine phosphorylation of signal transducer and activator of transcription (STAT1) 1^13^. STAT1 is phosphorylated on tyrosine 701, undergoes dimerization, translocates to the nucleus and regulates gene expression by binding to IFN-γ-activated sequence elements in the promoters of IFN-γ-regulated genes. Based on studies in Thailand and Taiwan, high-titers of anti-IFN-γ autoAb^7^ are present in 81% of patients with NTM (52 patients) and 96% of patients with other opportunistic infections with or without NTM (45 patients); neutralizing activity of these autoAbs was suggested as a parameter for monitoring of disease onset and progression. Until now, detection of anti-IFN-γ autoAbs is developed^7,14^ but is not widely used in clinical studies. This study aimed to measure anti-IFN-γ autoAbs in patients with NTM infection. We also investigated the blocking effect of anti-IFN-γ autoAbs on IFN-γ signaling pathway, especially on STAT1-IRF transactivation and its bioactivity including inflammatory response and cytokines production.

## Results

### Creating modified sandwich ELISA to measure anti-IFN-γ autoAbs in patients with NTM infection

**Figure 1:**
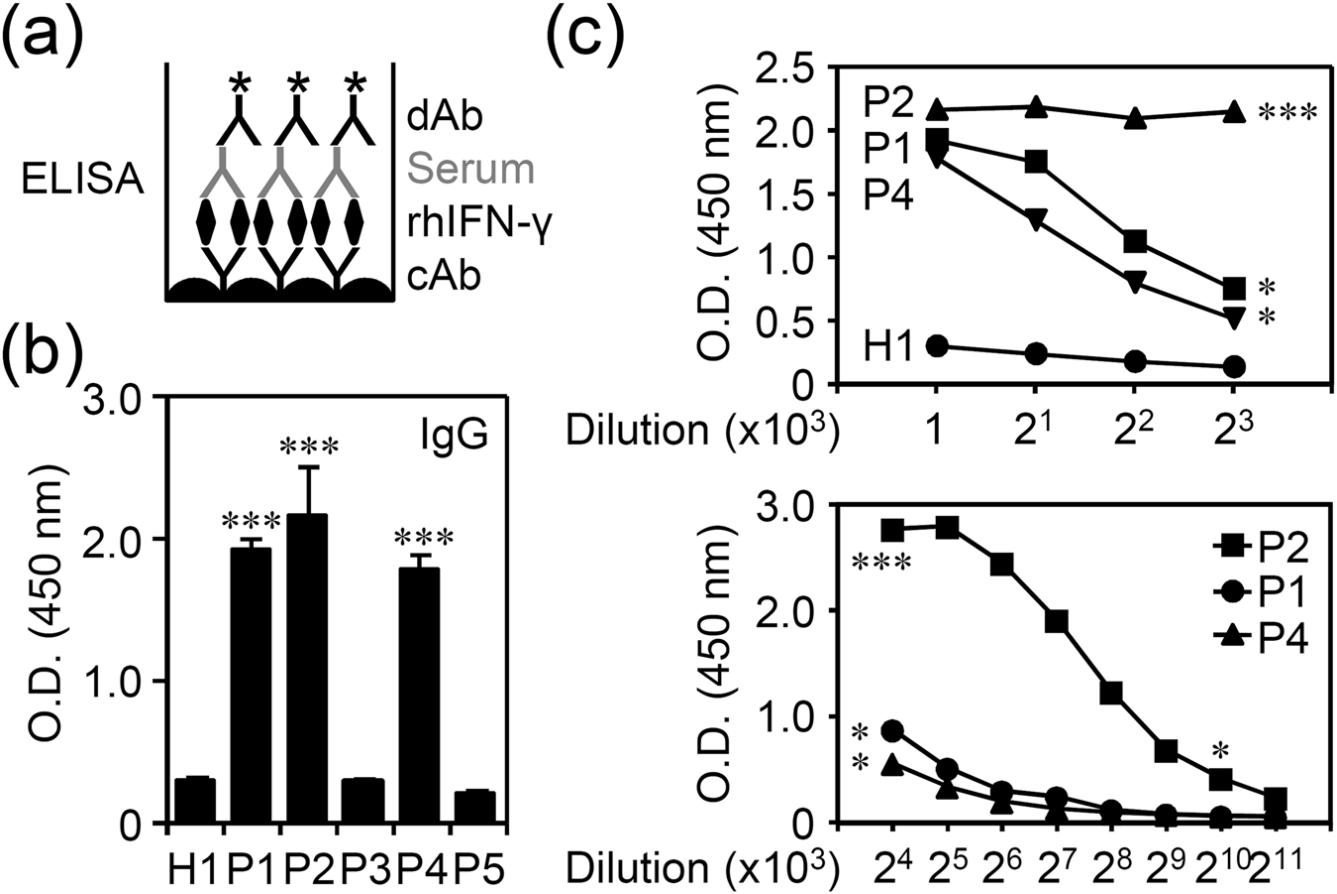
Measuring anti-IFN-γ autoAbs by colorimetric ELISA. (**a**) The protocol of ELISA for measuring anti-IFN-γ autoAbs has been modified from conventional IFN-γ ELISA assay by coating with capture Ab (cAb) and adding recombinant human IFN-γ (rhIFN-γ) and tested serum followed by adding detection Ab (dAb). (**b**) High production of anti-IFN-γ autoAbs in tested patient sera (P1, P2, and P4). (**c**) Measuring anti-IFN-γ autoAbs at various dilutions of patient serum as indicated. H1, healthy control 1. Quantitative data (optical density, O.D.) are shown as mean ± SD from three independent experiments. ^*^p < 0.05 and ^***^p < 0.001, compared with H1.

Assay of anti-IFN-γ autoAbs is required to confirm adult-onset immunodeficiency; however, the methods are not used on a routine basis. Generation of anti-IFN-γ autoAbs can be detected by ELISA^10^ and Luciferase Immunoprecipitation System^7^. In this study, a modified colorimetric sandwich ELISA was developed by using a conventional and commercially available IFN-γ sandwich ELISA kit to measure anti-IFN-γ autoAbs in the serum of patients with NTM infection. Collection and preparation of tested sera are described in Materials and Methods. In brief, a total of five patient clinically diagnosed with recurrent opportunistic infection and NTM patients without HIV infection were enrolled for testing the generation and the neutralizing activity of anti-IFN-γ autoAbs. The protocol of modified sandwich ELISA is summarized in Fig. 1a and described in Materials and Methods. In contrast to a conventional IFN-γ sandwich ELISA kit, we added recombinant human IFN-γ as a capture antigen to measure the presence of anti-IFN-γ autoAbs. Compared with representative healthy control, production of anti-IFN-γ autoAbs was significantly (*p* < 0.001) increased in 3 out of 5 serum samples of the patients including P1, P2, and P4, at 1:1000 dilution of tested sera (Fig. 1b and S1). Titration of P1 and P4 sera showed a decrease in the levels of anti-IFN-γ autoAbs at 2^3^x10^3^ dilutions, while P2 showed a decrease in the levels of anti-IFN-γ autoAbs at 2^10^ × 10^3^ dilutions (Fig. 1c). The data indicate that a modified sandwich ELISA-based method can be used to measure anti-IFN-γ autoAbs.

### Performing immunoblotting to confirm the detection of anti-IFN-γ autoAbs

**Figure 2:**
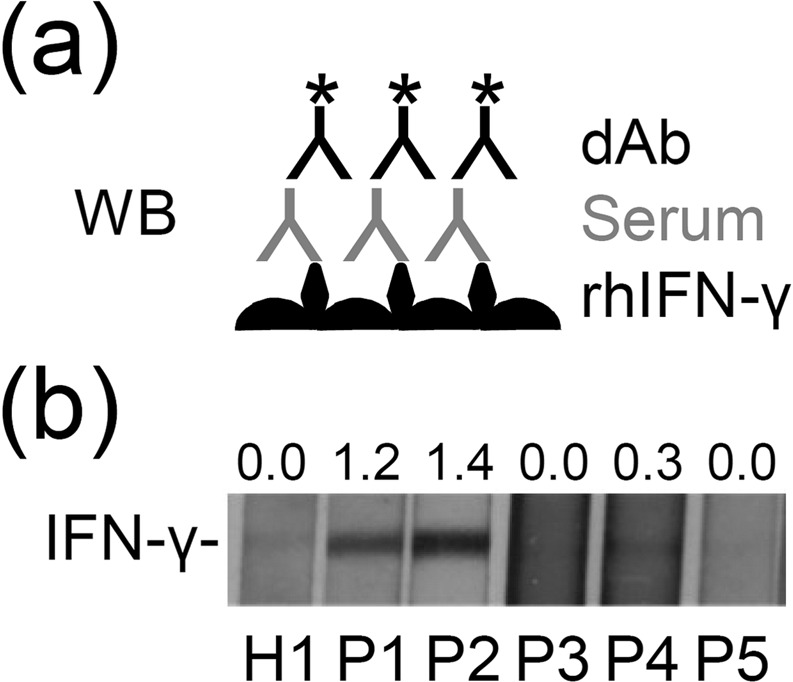
Measuring anti-IFN-γ autoAbs by immunoblot analysis. (**a**) The protocol of Western blot (WB)-based analysis for measuring anti-IFN-γ autoAbs has been modified by loading recombinant human IFN-γ (rhIFN-γ) through a polyacrylamide gel for protein separation and then the protein was transferred to a PVDF membrane. After blocking with BSA and incubating with patient serum, detection Ab (dAb) was added. (**b**) Representative immunoblot showing high binding activity of anti-IFN-γ autoAbs in tested patient sera (P1, P2, and P4). Relative density of measured proteins is also shown. Full-length blots/gels are presented in Supplementary Figure 1.

We have detected the presence of anti-IFN-γ autoAbs in serum of patients with NTM infection by a modified sandwich ELISA. To confirm the results, we used standard Western blotting to detect anti-IFN-γ autoAbs. Recombinant human IFN-γ protein was separated through a polyacrylamide gel and then transferred to a PVDF membrane with a minor protocol modification as described in legend to Fig. 2a. After blocking with bovine serum albumin (BSA), the membrane was cut and incubated with individual tested sera. Results of immunoblot were similar to the data of modified ELISA-based assay confirming the presence of anti-IFN-γ autoAbs in P1, P2, and P4 but not in healthy control, P3, and P5 (Fig. 2b and S2). The results illustrate application of immunoblotting for assay of anti-IFN-γ autoAbs in the tested sera.

### The expression of IFN-γ, TNF-α, and IL-2 in patients with NTM infection

**Figure 3:**
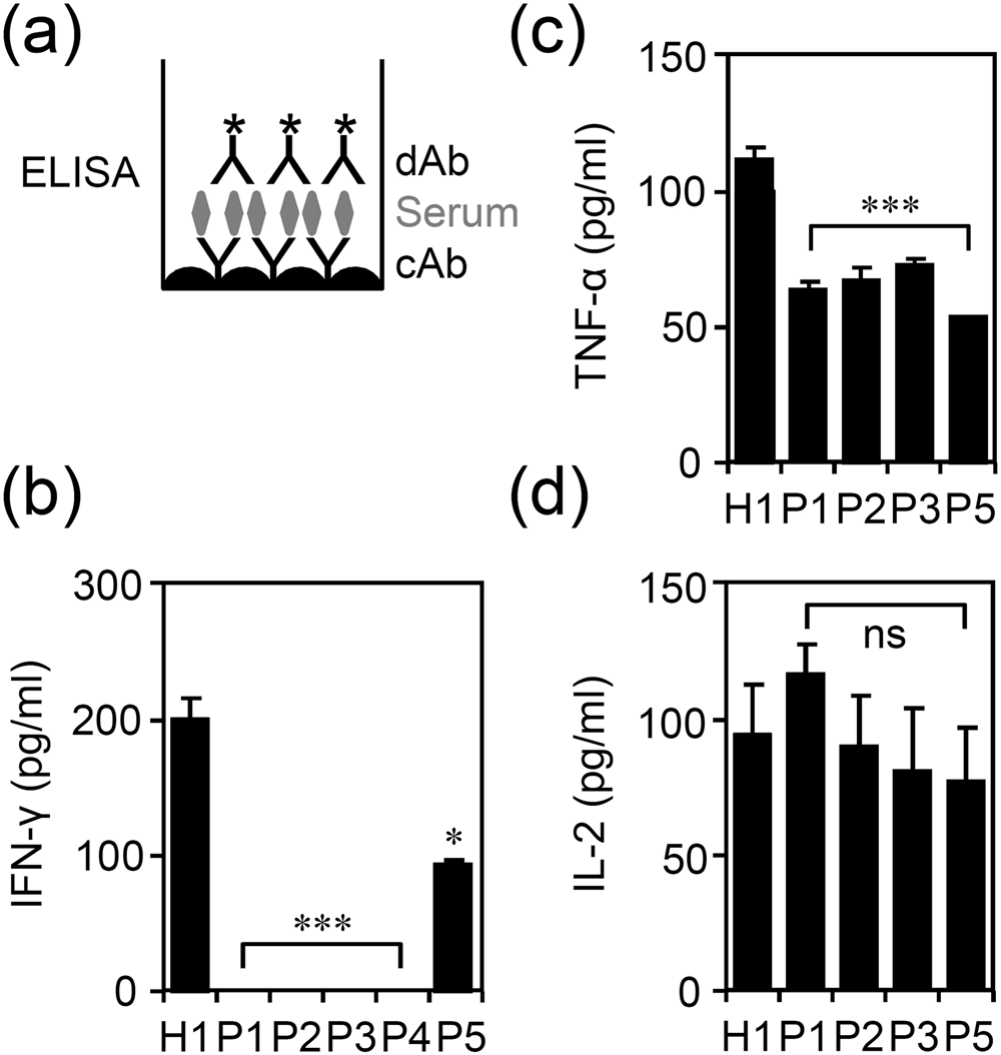
Detection of IFN-γ expression by colorimetric ELISA. (**a**) Conventional IFN-γ detection ELISA was performed to measure IFN-γ. Quantitative data (optical density, O.D.) are shown as mean ± SD of three independent experiments; ns, not significant. Measuring IFN-γ (**b**), TNF-α (**c**), and IL-2 (**d**) expression in tested serum; data are mean ± SD of three independent experiments. ^*^p < 0.05 and ^***^p < 0.001, compared with H1.

Expression of IFN-γ is highly related to T cell activation in response to antigen stimulation as well as inflammatory activation^1–3^. To verify the effects of anti-IFN-γ autoAbs on circulating cytokines related to T cell activation such as IFN-γ, TNF-α, and IL-2, we examined the level of cytokine expression in all tested sera. A commercial sandwich IFN-γ, TNF-α, and IL-2 ELISA was used as summarized in Fig. 3a. The results show a significant decrease in the level of serum IFN-γ (Fig. 3b) and TNF-α (Fig. 3c) (p < 0.05) in all tested patient sera compared with healthy control. No further difference was shown in IL-2 expression (Fig. 3d). In the sera of P1, P2, and P4, IFN-γ could not be measured (*p* < 0.001). According to the data, patients with NTM infection demonstrate a significant decrease in IFN-γ and TNF-α in the tested sera.

### Binding activity of synthetic IFN-γ peptide (amino acid residues 121–131) to anti-IFN-γ autoAb in patients with NTM infection

**Figure 4:**
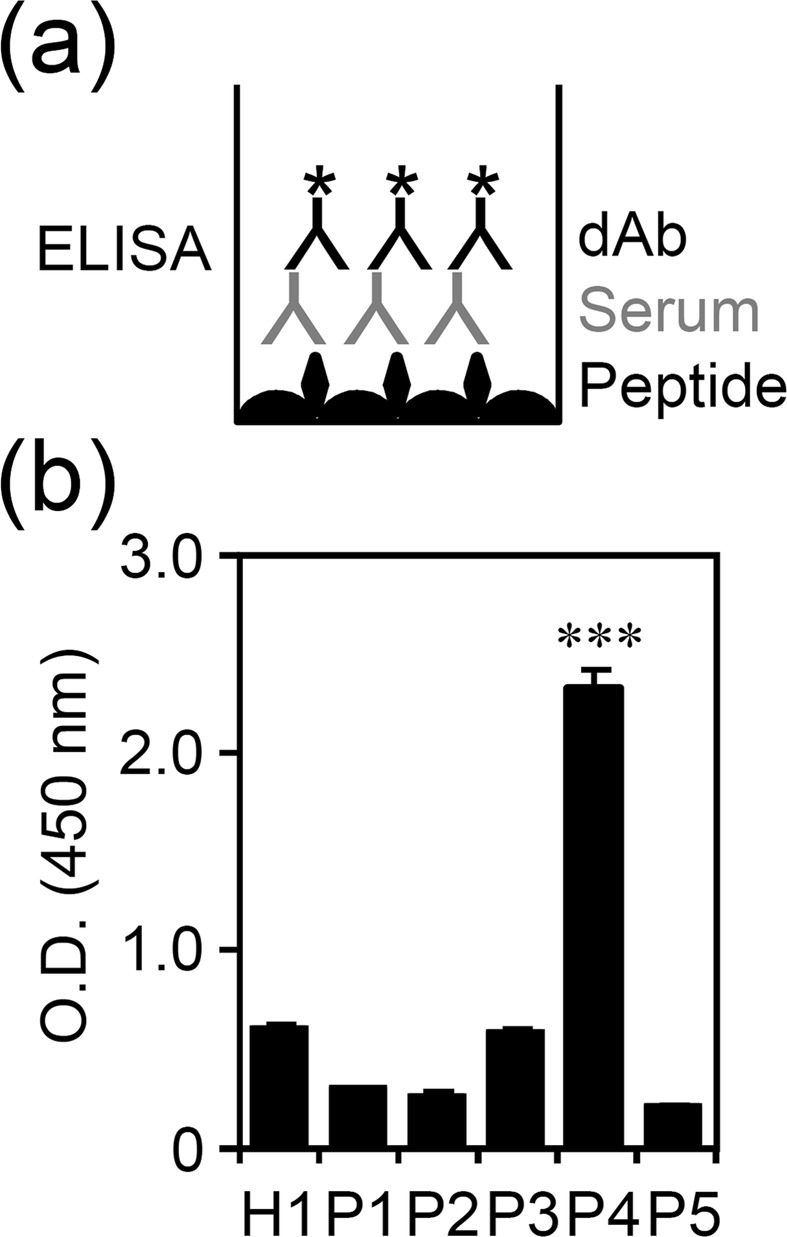
Measuring anti-IFN-γ (amino acid 121–131) autoAbs by colorimetric ELISA. (**a**) The protocol of direct ELISA for measuring anti-IFN-γ amino acids 121–131 autoAbs has been modified by coating with synthetic peptide (IFN-γ amino acid 121–131) and adding 10^3^ dilution of tested serum followed by adding detection Ab (dAb). (**b**) High production of anti-IFN-γ autoAbs in tested patient serum (P4). Quantitative data (optical density, O.D.) are shown as mean ± SD of three independent experiments. ^***^p < 0.001, compared with H1.

Lin and colleagues recently found a major epitope of anti-IFN-γ autoAbs targeting IFN-γ at amino acid residues 121–131, a region crucial for IFN-γR activation^12^. A direct ELISA summarized in Fig. 4a was performed. Microplate was coated with synthetic IFN-γ peptide (amino acid residues 121–131) and tested sera were added followed by incubation with detecting Abs. The results were not consistent with the data of sandwich ELISA (Fig. 1b**)** since only P4 serum showed a significantly (*p* < 0.001) higher binding to anti-IFN-γ autoAbs (Fig. 4b). The data demonstrate variability in reactivity of anti-IFN-γ autoAbs with potential epitope of IFN-γ.

### Anti-IFN-γ autoAbs inhibit IFN-γ-induced activation of STAT1/IRF-1 signaling

**Figure 5:**
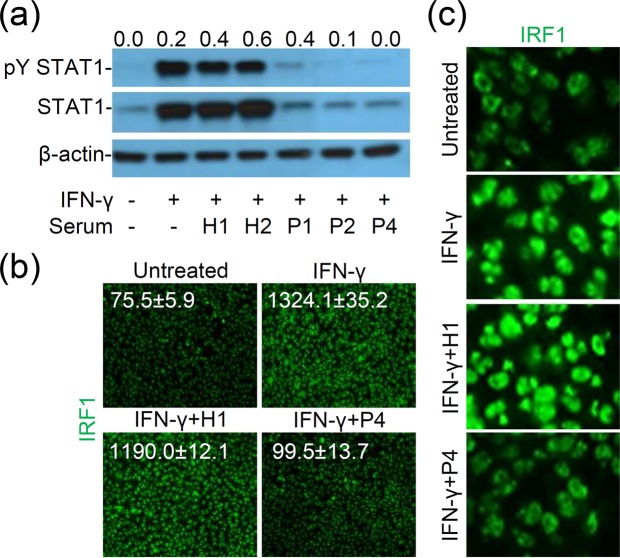
Anti-IFN-γ autoAbs impair IFN-γ-induced STAT1 phosphorylation/expression and IRF1 expression/nuclear translocation. (**a**) Representative Western blotting shows blockade of IFN-γ (10 ng/ml)-induced STAT1 phosphorylation at Tyr701 (pY STAT1) and STAT1 protein expression by 10^3^ dilution of anti-IFN-γ autoAbs-contain patient sera (P1, P2, and P4) in human monocytic THP-1 cells incubated for 6 h. Relative ratio of measured proteins (pY STAT1/STAT1) are also shown. H, healthy control. Full-length blots/gels are presented in Supplementary Figure 2. Immunostaining followed by fluorescence microscopic observation was used to determine IRF1 expression (**b**) and nuclear translocation (**c**) in the indicated cells stimulated with IFN-γ (10 ng/ml) for 6 h with or without tested serum. Data are shown as a representative image.

Previous studies^7,12^ by using flow cytometry showed that STAT1 phosphorylation is blocked by anti-IFN-γ autoAbs. In this study, we used Western blot analysis to check neutralizing activity of anti-IFN-γ autoAbs against IFN-γ-induced STAT1 phosphorylation and protein induction. Compared with anti-IFN-γ autoAb-negative healthy controls, anti-IFN-γ autoAb-positive sera of NTM patients (P1, P2, and P4) effectively decreased either phosphorylation of STAT1 at tyrosine 701 or STAT1 protein expression (Fig. 5a and S3). Fluorescent immunostaining showed an increase in IRF-1 expression caused by IFN-γ stimulation while anti-IFN-γ autoAb-positive patient serum P4 effectively decreased this enhancement (Fig. 5b). Further observations showed that anti-IFN-γ autoAbs blocked IFN-γ-induced nuclear translocation of IRF1 (Fig. 5c). These results illustrate the neutralizing activity of anti-IFN-γ autoAbs against IFN-γ-induced activation of STAT1/IRF-1 signaling.

### Anti-IFN-γ autoAbs block nitrite and chemokine production in IFN-γ-activated monocytes and cytokine production in anti-CD3/CD28-activated T cells

**Figure 6:**
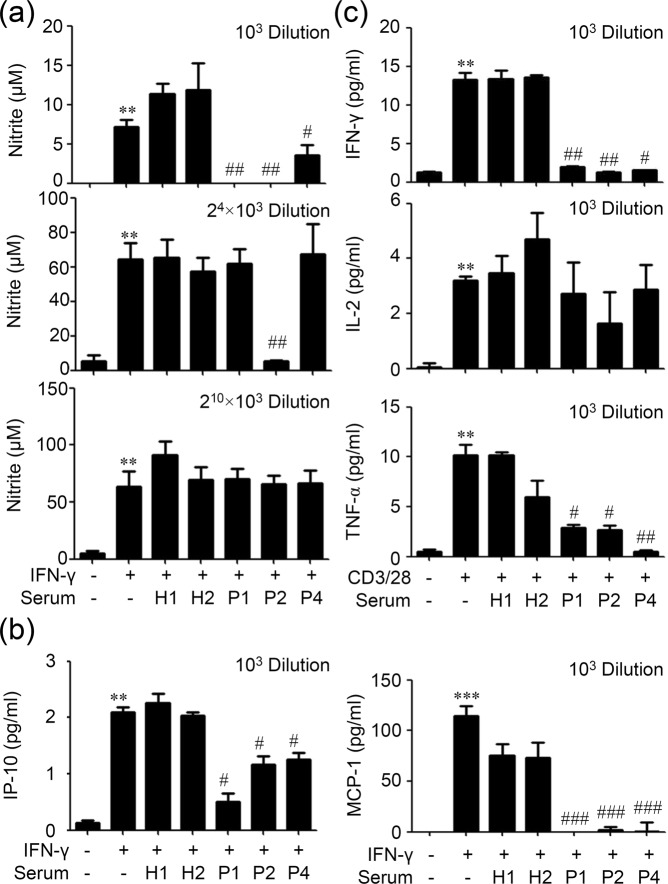
Anti-IFN-γ autoAbs block nitrite and chemokine production in monocytes and cytokine production during T cell activation. (**a**) Griess reaction showed the blockade of IFN-γ (10 ng/ml)-induced nitrite production by the indicated dilution of anti-IFN-γ autoAbs-contain patient sera (P1, P2, and P4), in human monocytic THP-1 cells incubated for 24 h. (**b**) ELISA showed the blockade of IFN-γ (10 ng/ml)-induced chemokine (IP-10 and MCP-1) production by 10^3^ dilution of anti-IFN-γ autoAbs-contain patient sera (P1, P2, and P4), in human monocytic THP-1 cells incubated for 24 h. (**c**) Data on IFN-γ, IL-2, and TNF-α production in anti-CD3/CD28 (0.5 μg/ml)-stimulated human Jurkat T cells incubated for 24 h. H, healthy control. The quantitative data are shown as mean ± SD of three independent experiments. ^**^p < 0.01 and ^***^p < 0.001, compared with untreated. ^#^*p* < 0.05, ^##^*p* < 0.01, and ^###^*p* < 0.001, compared with H1.

IFN-γ induces iNOS expression to promote nitric oxide and chemokine production triggering inflammation^1^ and enhancing microbicidal ability. By using an inflammatory cell model of IFN-γ-induced activation in human monocytic THP-1 cells, production of nitric oxide and chemokines was monitored in the presence or absence of anti-IFN-γ autoAb-positive patient serum. Compared with healthy controls, 10^3^ dilution of patient serum significantly (*p* < 0.05) blocked IFN-γ-induced production of nitrite (Fig. 6a, upper) and chemokines IP-10 and MCP-1 (Fig. 6b). According to the results of Fig. 1c, 2^4^ × 10^3^ dilutions were used to test its neutralizing titer against IFN-γ-induced nitrite production while patient 2 still has a higher titer of anti-IFN-γ autoAbs. Results showed that 2^4^ × 10^3^ dilution of patient serum 1 and 4 lost its neutralizing effect on IFN-γ-induced nitrite production (Fig. 6a, middle); however, patient serum 2 still has significant (*p* < 0.01) blocking activity until 2^10^ × 10^3^ dilution (Fig. 6a, lower). The results indicate the neutralizing activity of anti-IFN-γ autoAbs present in patients’ serum and a higher titer of anti-IFN-γ autoAbs displays a higher neutralizing activity. Alternatively, in an anti-CD3/CD28-activated human Jurkat T cell model, we verified the blocking effects of anti-IFN-γ autoAbs against IFN-γ-regulated T cell cytokine production. ELISA showed that anti-IFN-γ autoAb-positive patient serum significantly (*p* < 0.001) blocked anti-CD3/CD28-induced production of cytokines including IFN-γ and TNF-α but had no effect on IL-2 (Fig. 6c). These results demonstrate the methods for testing the neutralizing activity of anti-IFN-γ autoAbs against IFN-γ-induced bioactivity in monocytes and T cells.

## Discussion

Generation of anti-IFN-γ autoAbs is tightly associated with adult-onset immunodeficiency in NTM infection^7,15^. For NTM, the first step in of the infection is overcoming the innate antimicrobial response provided by the macrophages and IFN-γ. In addition to the inherited mutations of IFN-γ signaling-associated factors, such as IFN-γR and STAT1, inhibiting IFN-γ either by reducing secretion from T and NK cells or by neutralization with anti-IFN-γ autoAbs impairs the ability of macrophages to kill intracellular mycobacteria^1,11^. Although detection of anti-IFN-γ autoAbs has been established, functional characterization of these anti-IFN-γ autoAbs is limited only to a routine assay of IFN-γ-activation of STAT1. In the presence of anti-IFN-γ autoAbs, we found a decrease in IFN-γ expression in peripheral blood of patients. This study provides several assays for detection and characterization of neutralizing activity of anti-IFN-γ autoAbs. By using enough sample size and well-designed controls, such as tuberculosis and latent tuberculosis infection, in addition to detecting anti-IFN-γ autoAbs, a functional neutralization assay can be used for screening the pathogenic titers of anti-IFN-γ autoAbs generated in NTM patients with adult-onset immunodeficiency. However, in patients without a positive in the detection of anti-IFN-γ autoAbs, the involvement of genetic defects on IFN-γ signaling is also needed to be validated by using genomic measurement.

In clinical practice, fast and accurate evaluation and examination of new patients are recommended for rapid diagnosis and immediate treatment and to improve diagnosis especially in patients with infectious diseases^16^. For NTM patients, detection of anti-IFN-γ autoAbs is needed for evaluation on clinical use. ELISA has the most common technique used to detect anti-IFN-γ autoAbs since 1989 in various diseases including HIV, non-HIV opportunistic infections, *Mycobacterium tuberculosis* infection, NTM infection of disseminated or non-disseminated type, and Salmonella infection^11^. Radioimmunoassay (RIA) has been used as well, but it is no longer a common procedure. In this study, we developed a modified sandwich ELISA to measure anti-IFN-γ autoAbs. We modified the method by adding recombinant human IFN-γ protein before incubation with the tested serum. Number of our observations is limited to make a conclusion for evaluating the sensitivity of the method regarding sample size is rare as well as due to a few control groups; however, specificity of ELISA-based detection was confirmed by conventional Western blot analysis. Ideally, commercial kit ELISA should facilitate the process. On the other hand, Western blotting remains cheaper but more laborious process due to various buffer solutions and their recipes and susceptibility to human error as well as troubleshooting electrical stability^17^.

The study has demonstrated that a potential epitope target of IFN-γ (amino acids 121–131) can interact with anti-IFN-γ autoAbs^12^. Since this region plays a crucial role in IFN-γR activation, it is speculated to account for neutralizing activity of anti-IFN-γ autoAbs against IFN-γ-induced signaling and bioactivity. Amino acid sequence of this epitope is highly homologous to a stretch in the Noc2 protein of *Aspergillus spp*. which cross-reacted with autoAbs from the patients. Rats immunized with *Aspergillus* Noc2 developed Abs that reacted with human IFN-γ in that study. A molecular mimicry-based autoimmunity was hypothesized to be an important facilitator of immunopathogenesis of adult-onset immunodeficiency. By using direct ELISA, we assayed binding activity of the identified target epitope to anti-IFN-γ autoAbs, and the results showed variable patterns among the patients. Thoroughly, the use of different source of recombinant IFN-γ and the variants of the binding epitopes needs further validation in the near future.

Genes involved in antimicrobial activity are specifically induced by STAT1/IRF1-mediated transactivation. In this study, we demonstrated that anti-IFN-γ autoAbs in adult-onset immunodeficiency patient serum can block STAT1/IRF1 activation. Its downstream targets and effects, including iNOS/NO biosynthesis and production of chemokines MCP-1 and IP-10, are blocked by anti-IFN-γ Abs in activated monocytes. Furthermore, in activated T cells, secreted IFN-γ plays immunomodulatory effects on T cell cytokine production. We also showed that anti-IFN-γ autoAbs inhibit cytokine production including IFN-γ and TNF-α during T cell activation. As similar with a decrease in serum levels of IFN-γ and TNF-α expression in patients, the blockade effects of anti-IFN-γ Abs are possibly not only on IFN-γ neutralization but also on T cell-derived TNF-α production. These results indicate that generation of anti-IFN-γ autoAbs in patients with adult-onset immunodeficiency has immunosuppressive effects in innate and adaptive immunity and may block antibacterial effects in NTM infection.

For the demonstration of the pathogenic anti-IFN-γ autoAbs involved in patients with adult-onset immunodeficiency, several tests should be addressed by detecting (1) the titer of anti-IFN-γ autoAbs recognizing the target epitope(s), (2) the neutralizing/blocking effects on IFN-γ signaling and bioactivities, and (3) the involvement of genetic mutations on IFN-γ signaling pathways. In conclusion, our study illustrates variability in patterns of antibody-antigen reactivity among the tested cases demonstrated by a modified sandwich ELISA-based colorimetric assays and Western blot analysis. We also developed several methods for characterization of the neutralizing activity. Monitoring the neutralizing and/or pathogenic titers of anti-IFN-γ autoAbs may be important for diagnosis and prognosis of disease progression and for verification of efficacy of immune therapy. In this study, the downstream signals and bioactivities of IFN-γ were selectively evaluated. In the presence of neutralizing anti-IFN-γ autoAbs, IFN-γ-induced immunomodulation, antimicrobial, and anticancer activities are therefore speculated to be blocked. This issue needs a comprehensive study methodology by using genomic and proteomic approaches. Further studies need to investigate the role of anti-IFN-γ autoAbs that affect biological function of IFN-γ such as its antimicrobial activity.

## Methods

### Ethics statement

Human study of this project was performed according to guidelines established by the Taipei Medical University (TMU)-Joint Institutional Review Board (TMU-JIRB. An informed consent from all participants as approved by TMU-JIRB was obtained.

### Serum

As approved by TMU-JIRB (N201804015), five patients individually admitted to the Division of Infectious Diseases at Taipei Medical University affiliated Hospitals were enrolled in the study with clinical diagnosis including disseminated infections with opportunistic pathogens and NTM infections, such as *Mycobacterium kansasii* and *Mycobacterium abscessus*, and the patients were HIV-negative according to the criteria of adult-onset immunodeficiency (https://rarediseases.info.nih.gov/diseases/11992/adult-onset-immunodeficiency-with-anti-interferon-gamma-autoantibodies). For each patient, basic demographic data, medical history, physical examination data, and subsequent progress were recorded on a standard data form. Five healthy individuals were also enrolled to collect blood samples as controls. Serum was separated and prepared by using a conventional method as described elsewhere (https://www.thermofisher.com/tw/zt/home/references/protocols/cell-and-tissue-analysis/elisa-protocol/elisa-sample-preparation-protocols/plasma-and-serum-preparation.html) and serum samples were stored at −80 °C until use.

### Reagents and Abs

The recombinant human cytokine IFN-γ was purchased from PeproTech (Rocky Hill, NJ). Other reagents used in this study were obtained from Sigma-Aldrich (St Louis, MO). Synthetic IFN-γ peptide (amino acid residue 121–131) was obtained from Kelowna International Scientific (Taipei, Taiwan) as reported previously^12^. No drug treatments used in this study caused cell cytotoxicity. The antibodies used included those generated in rabbits and directed against human STAT1 Tyr701, STAT1, and IFN regulatory factor (IRF) 1 (Cell Signaling Technology, Beverly, MA); a mouse mAb specific for β-actin (Sigma-Aldrich); and polyclonal anti-rabbit Atg8 (LC3) I/II (MBL international). Alexa Fluor 488- and horseradish peroxidase (HRP)-conjugated goat anti-rabbit and anti-human IgG were obtained from Chemicon International (Temecula, CA) and Jackson ImmunoResearch laboratories, Inc, (West Grove, PA).

### Cell culture

Human monocytic THP-1 cells (ATCC, TIB-202) and human Jurkat T cells (ATCC, TIB-152) were grown and maintenance on 10 cm plate in RPMI Medium 1640 (RPMI; Invitrogen Life Technologies, Rockville, MD), with L-glutamine and supplemented with 10% heat-inactivated fetal bovine serum (FBS; Invitrogen Life Technologies), 50 units of penicillin, and 50 mg/ml of streptomycin. All of cells were maintained in a humidified atmosphere with 5% CO_2_ and 95% air.

### Modified ELISA

Sandwich and direct ELISA-based approaches were used to detect anti-IFN-γ autoAbs and Abs against synthetic peptide in the study. For anti-IFN-γ autoAb detection, a commercial human IFN-γ sandwich ELISA kit (R&D Systems, Minneapolis, MN) was modified according to the manufacturer instructions. In brief, we coated microwells with capture Abs against IFN-γ, blocked the wells with 0.5% bovine serum albumin (BSA) in PBS Tween 20 buffer, added recombinant human IFN-γ and tested sera, and then developed with HRP-conjugated detecting Abs against human IgG. For assay of Abs against synthetic peptide, we coated the wells with peptide and blocked with BSA. We added tested serum and then developed the signal with HRP-conjugated detection Abs against human IgG. Relative optical density (O.D.) is shown.

### Immunoblotting

Immunoblotting procedures are described elsewhere^18^. Briefly, recombinant human IFN-γ or total lysates were separated using SDS-polyacrylamide gel electrophoresis and then transferred to a polyvinylidene difluoride membrane (Millipore Corporation, Billerica, MA). After blocking with 5% BSA, cut blots were developed with one thousand dilution of tested serum and the indicated primary Abs. Finally, the blots were hybridized with HRP-conjugated goat anti-human or anti-rabbit IgG and developed using an ECL western blot detection kit (Millipore Corporation). The relative signal intensity of the proteins was quantified using ImageJ software (version 1.41o; W. Rasband, National Institutes of Health, Bethesda, MD, USA).

### Immunostaining

Cells were fixed with 4% paraformaldehyde, permeabilized with 0.5% Triton X-100, and washed twice with ice-cold PBS. Cells were stained with Abs against pY-STAT1 and STAT1 followed by a staining of Alexa 488-conjugated goat anti-human IgG. Cells were visualized under a fluorescent microscope (BX51; Olympus, Tokyo, Japan).

### Monocyte and T cell activation

For THP-1 cell activation, recombinant human IFN-γ (10 ng/ml) was used to stimulate monocytes according to our previous studies^19^. For Jurkat T cell activation, the procedures are described elsewhere^20^. We used anti-CD3 precoated 24-well culture plate (0.5 µg/well) and anti-CD28 was added to the culture medium (0.5 µg/ml). Cells (10^6^/ml) were co-incubated with diluted tested serum (1:1,000) and incubated at 37 °C in 5% CO_2_ at 100% humidity for 24 h. The supernatants were harvested for cytokine detection.

### Cytokine/chemokine detection

The concentration of IP-10, MCP-1, IFN-γ, IL-2, and TNF-α in cell-conditioned culture medium and serum are determined using ELISA kits (RD System) according to the manufacturer instruction.

### Griess reaction

The production of nitric oxide (NO) was assessed as accumulation of nitrite (NO^2−^) in the medium using a colorimetric reaction with Griess reagent according to our previous procedures^18^.

### Statistical analysis

Values are expressed in mean ± standard deviation (SD). Groups are compared by using Student’s two-tailed unpaired t test or one way ANOVA analysis followed by Dunnet post hoch test, as appropriate. These analyses are performed by using GraphPad Prism 4 software (GraphPad Software, La Jolla, CA). Statistical significance is set at *p* < 0.05.

## Supplementary information

Dyah et al Supplementary Information
