# Supplementary material for: Functional neutralization of anti-IFN-γ autoantibody in patients with nontuberculous mycobacteria infection
Source: Sci Rep. 2019 Apr 5;9:5682. doi: 10.1038/s41598-019-41952-1 (PMC6450904; doi:10.1038/s41598-019-41952-1)

Functional neutralization of anti-IFN-γ autoantibody in patients with nontuberculous mycobacteria infection

Dyah Ika Krisnawati[1,2,3], Yung-Ching Liu[4,5], Yuarn-Jang Lee[6], Yun-Ting Wang[2], Chia-Ling Chen[7], Po-Chun Tseng[2], Chiou-Feng Lin[1,2,*]

[1]Graduate Institute of Medical Sciences, College of Medicine, Taipei Medical University, Taipei 110, Taiwan; [2]Department of Microbiology and Immunology, School of Medicine, College of Medicine, Taipei Medical University, Taipei 110, Taiwan; [3]Dharma Husada Nursing Academy, Kediri, East Java, Indonesia; [4]Department of Internal Medicine, School of Medicine, College of Medicine, Taipei Medical University, Taipei 110, Taiwan; [5]Division of Infectious Diseases, Department of Internal Medicine, Shuang Ho Hospital, Taipei Medical University, Taipei 110, Taiwan; [6]Division of Infectious Diseases, Department of Internal Medicine, Taipei Medical University Hospital, Taipei 110, Taiwan; [7]Department of Respiratory Therapy, College of Medicine, Taipei Medical University, Taipei 110, Taiwan

Supplemental figure legends

Figure S1 Generation of anti-IFN-γ autoAb in patients with NTM infection. A modified ELISA was used to measure anti-IFN-γ autoAbs in tested control (n=5) and patient sera (n=5). The raw data (optical density, O.D.) in a box-whisker plot was shown.

Figure S2 Protein assay for Figure 2.

Figure S3 Protein assay for Figure 5.

Dyah et al Figure S1

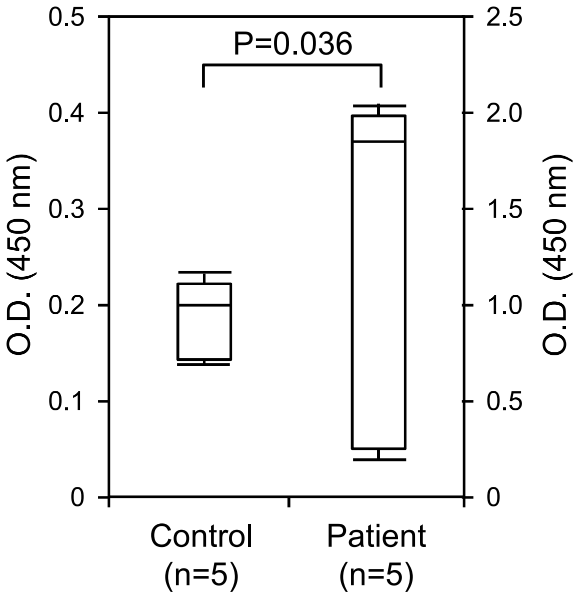

Dyah et al Figure S2

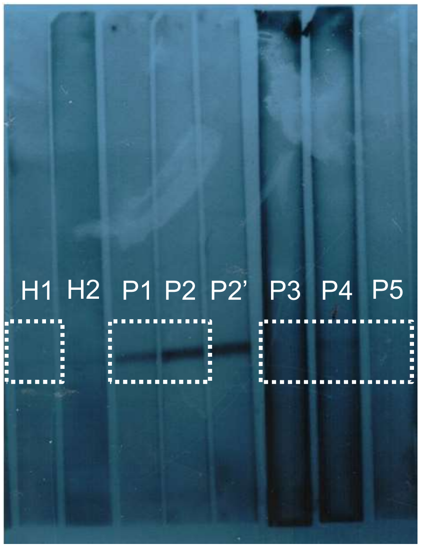

Dyah et al Figure S3

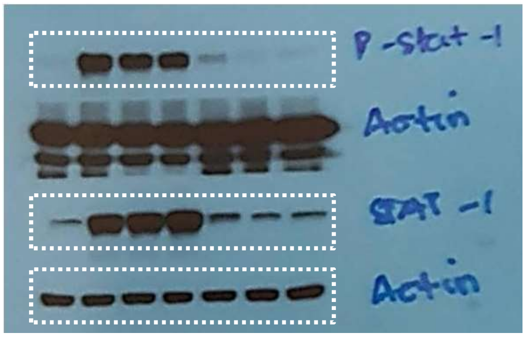

Supplement: Supplementary file 1 — Dyah et al Supplementary Information [file 41598_2019_41952_MOESM1_ESM.pdf]
